# Supplementary material for: Global chromatin conformation differences in the Drosophila dosage compensated chromosome X
Source: Nat Commun. 2019 Nov 25;10:5355. doi: 10.1038/s41467-019-13350-8 (PMC6877619; doi:10.1038/s41467-019-13350-8)
Supplement: Supplementary file 1 — Supplementary Information [file 41467_2019_13350_MOESM1_ESM.pdf]

Global chromatin conformation differences in the Drosophila dosage compensated chromosome X

Koustav Pal, Mattia Forcato, Daniel Jost, Thomas Sexton, Cédric Vaillant, Elisa Salviato, Emilia Maria Cristina Mazza, Enrico Lugli, Giacomo Cavalli, Francesco Ferrari

HiCPipe (Yaffe Tanay et al. [1])

Sample	Replicate	Total Pairs	Discarded Pairs	Kept Pairs
Male	A	639,467,382	598,536,196	40,931,186
Male	B	535,378,751	505,398,740	29,980,011
Male	Merge	1,174,846,133	1,103,934,936	**70,911,197**
Female	A	525,066,921	476,167,507	48,899,414
Female	B	573,378,931	497,288,017	76,090,914
Female	Merge	1,098,445,852	973,455,524	**124,990,328**

HiCLib (Imakaev et al. [2])

Sample	Replicate	Total Pairs	Discarded Pairs	Kept Pairs
Male	A1	160,609,800	146,322,053	14,287,747
Male	A2	158,119,428	144,025,206	14,094,222
Male	A3	160,559,337	146,270,219	14,289,118
Male	A4	160,178,817	145,910,435	14,268,382
Male	B1	177,854,486	161,473,733	16,380,753
Male	B2	176,891,839	160,551,750	16,340,089
Male	B3	180,632,426	164,470,001	16,162,425
Male	Merge	1,174,846,133	1,069,023,397	**105,822,736**
Female	A1	175,074,039	153,986,896	21,087,143
Female	A2	175,040,306	153,974,981	21,065,325
Female	A3	174,952,576	153,885,812	21,066,764
Female	B1	190,498,730	157,274,570	33,224,160
Female	B2	189,735,995	156,545,059	33,190,936
Female	B3	193,144,206	159,953,270	33,190,936
Female	Merge	1,098,445,852	935,620,588	**162,825,264**

Supplementary Table 1 Alignment results for sex-sorted embryos Hi-C dataset (GEO database accession number GSE94115).

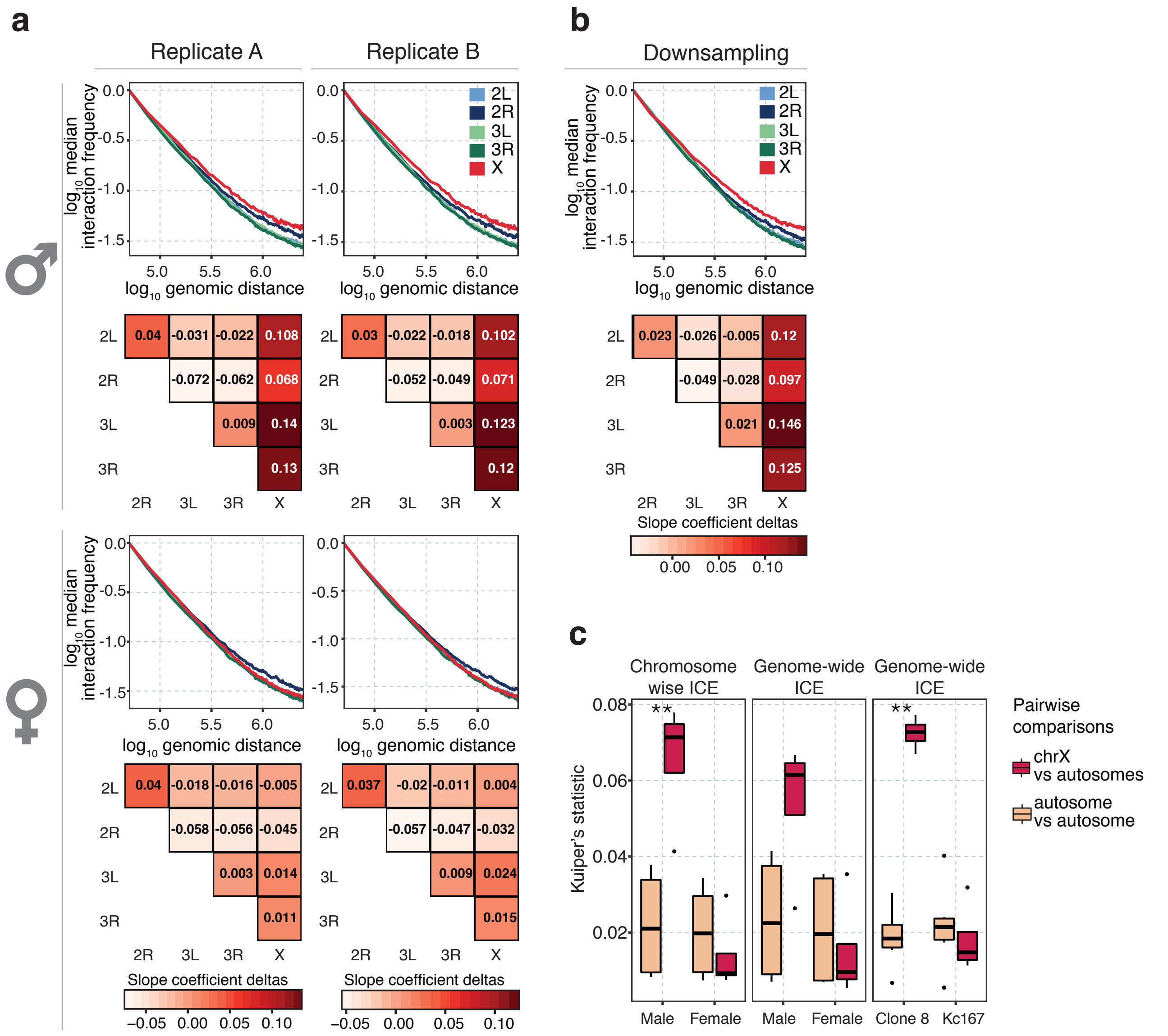

Supplementary Figure 1 - Differential interaction decay with distance is consistent across replicates and not affected by chrX copy number. (a) Interaction frequency decay with distance is shown in a log-log plot. Normalized (genome-wide ICE) Hi-C data binned at 25Kb were converted to contact frequencies as described in Methods for each replicate of the sex-sorted embryos dataset (Replicate A in left column, Replicate B in right column) for male (upper part) and female (lower part) samples. For any given genomic distance ($\log_{10}$ bp distance on x-axis) the median $\log_{10}$ Hi-C interaction frequency is reported (y-axis). Distances ranging from 50Kb (2 bins distance in the 25Kb bins matrix) to 2.5Mb are shown. The heatmaps below each plot shows the pairwise difference (deltas) of the rate of Hi-C signal decay (slope coefficient). The slope coefficients were computed considering distances <400Kb (≤5.6 in $\log_{10}$ distance). The colour scale and the numeric values report the differences between chromosomes indicated in the horizontal vs vertical axes labels. **(b)** Interaction frequency decay with distance is shown in a log-log plot. Normalized (chromosome-wise ICE) Hi-C data binned at 25Kb were converted to contact frequencies as described in Methods for the sex-sorted male sample (merged replicates) after reducing the coverage on autosomes by randomly sampling 50% of the aligned reads, to simulate the reduced coverage expected with copy number equal to chrX. All other analysis settings are the same as in panel a plots. The heatmaps below each plot

shows the pairwise difference (deltas) of the rate of Hi-C signal decay (slope coefficient) with the same settings as in panel “a” plots. **(c)** Boxplots of Kuiper's statistics are reported as alternative to assess the differences in rate of Hi-C decay grouped by autosomes and chrX in male and female embryos (left and centre) or cell lines (right). Alternative normalizations are also considered (genome-wide or chromosome-wide ICE, as indicated in each plot). Kuiper's statistic is computed as the sum of absolute values for the maximum positive and negative differences between the cumulative density functions (CDFs) of the interaction probability for autosomes or chrX. CDFs of interaction probability were estimated from 50Kb to 2.5Mb as cumulative sums of median Hi-C contact frequencies for each distance, then divided by the cumulative sum maximum value to make it equal to probability 1. For each box the median is marked as horizontal line, the boxes mark the interquartile range (IQR), the whiskers extend up to 1.5 IQR and individual data points are shown for outliers beyond this range.

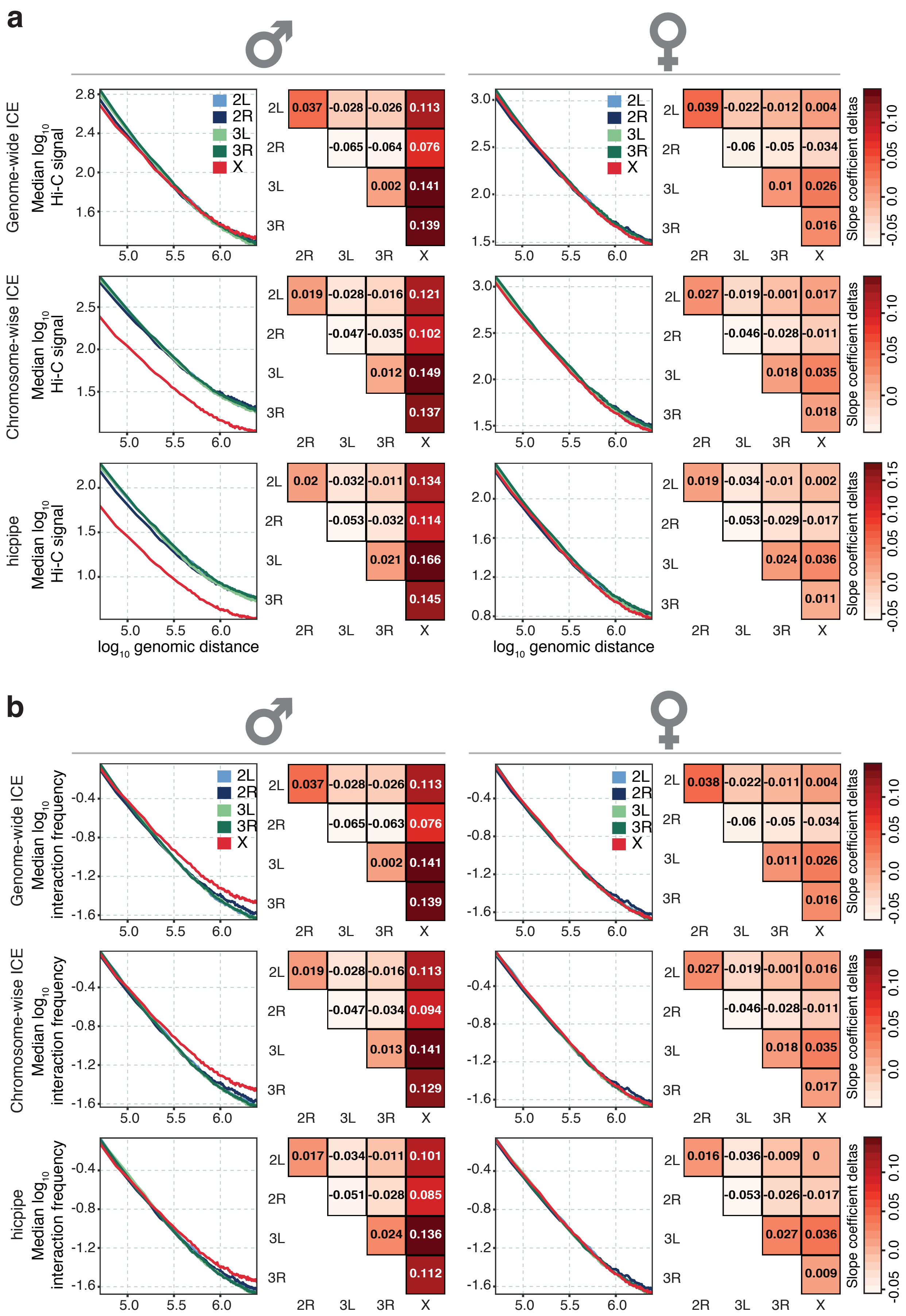
a
b
Genome-wide ICE
Chromosome-wise ICE
hicpipe
Median log10 Hi-C signal
Median log10 interaction frequency
log10 genomic distance
2L
2R
3L
3R
X
Slope coefficient deltas
0.037 -0.028 -0.026 0.113
-0.065 -0.064 0.076
0.002 0.141
0.139
0.039 -0.022 -0.012 0.004
-0.06 -0.05 -0.034
0.01 0.026
0.016
0.019 -0.028 -0.016 0.121
-0.047 -0.035 0.102
0.012 0.149
0.137
0.027 -0.019 -0.001 0.017
-0.046 -0.028 -0.011
0.018 0.035
0.018
0.02 -0.032 -0.011 0.134
-0.053 -0.032 0.114
0.021 0.166
0.145
0.019 -0.034 -0.01 0.002
-0.053 -0.029 -0.017
0.024 0.036
0.011
0.037 -0.028 -0.026 0.113
-0.065 -0.063 0.076
0.002 0.141
0.139
0.038 -0.022 -0.011 0.004
-0.06 -0.05 -0.034
0.011 0.026
0.016
0.019 -0.028 -0.016 0.113
-0.047 -0.034 0.094
0.013 0.141
0.129
0.027 -0.019 -0.001 0.016
-0.046 -0.028 -0.011
0.018 0.035
0.017
0.017 -0.034 -0.011 0.101
-0.051 -0.028 0.085
0.024 0.136
0.112
0.016 -0.036 -0.009 0
-0.053 -0.026 -0.017
0.027 0.036
0.009

Supplementary Figure 2 - ChrX specific difference in interaction decay with distance is robust to several variations of analysis parameters. (a) Interaction signal decay with distance is shown in a log-log plot. Hi-C data binned at 25Kb are shown for male (upper part) and female (lower part) samples of the sex-sorted embryos dataset, normalized with three alternative procedures: genome-wide ICE (left column), chromosome-wise ICE (central column) and hicpipe (right column). For any given genomic distance ($\log_{10}$ bp distance on x-axis) the median $\log_{10}$ Hi-C signal is reported (y-axis). Distances ranging from 50Kb (2 bins distance in the 25Kb bins matrix) to 2.5Mb are shown. The heatmaps beside each plot shows the pairwise difference (deltas) of the rate of Hi-C signal decay (slope coefficient). The slope coefficients were computed considering distances <400Kb (≤5.6 in $\log_{10}$ distance). The colour scale and the numeric values report the differences between chromosomes indicated in the horizontal vs vertical axes labels. **(b)** The same Hi-C data matrices as in panel a were processed by probabilistic transformation of interaction frequency as described in Giorgetti et al.[3], by dividing Hi-C contact frequencies by the mean Hi-C signal at the first informative diagonal (2 bins distance). Interaction probability decay with distance are then reported in a log-log plot using the same settings as in panel a. The heatmaps beside each plot shows the pairwise difference (deltas) of the rate of Hi-C signal decay (slope coefficient) with the same settings as in panel a plots.

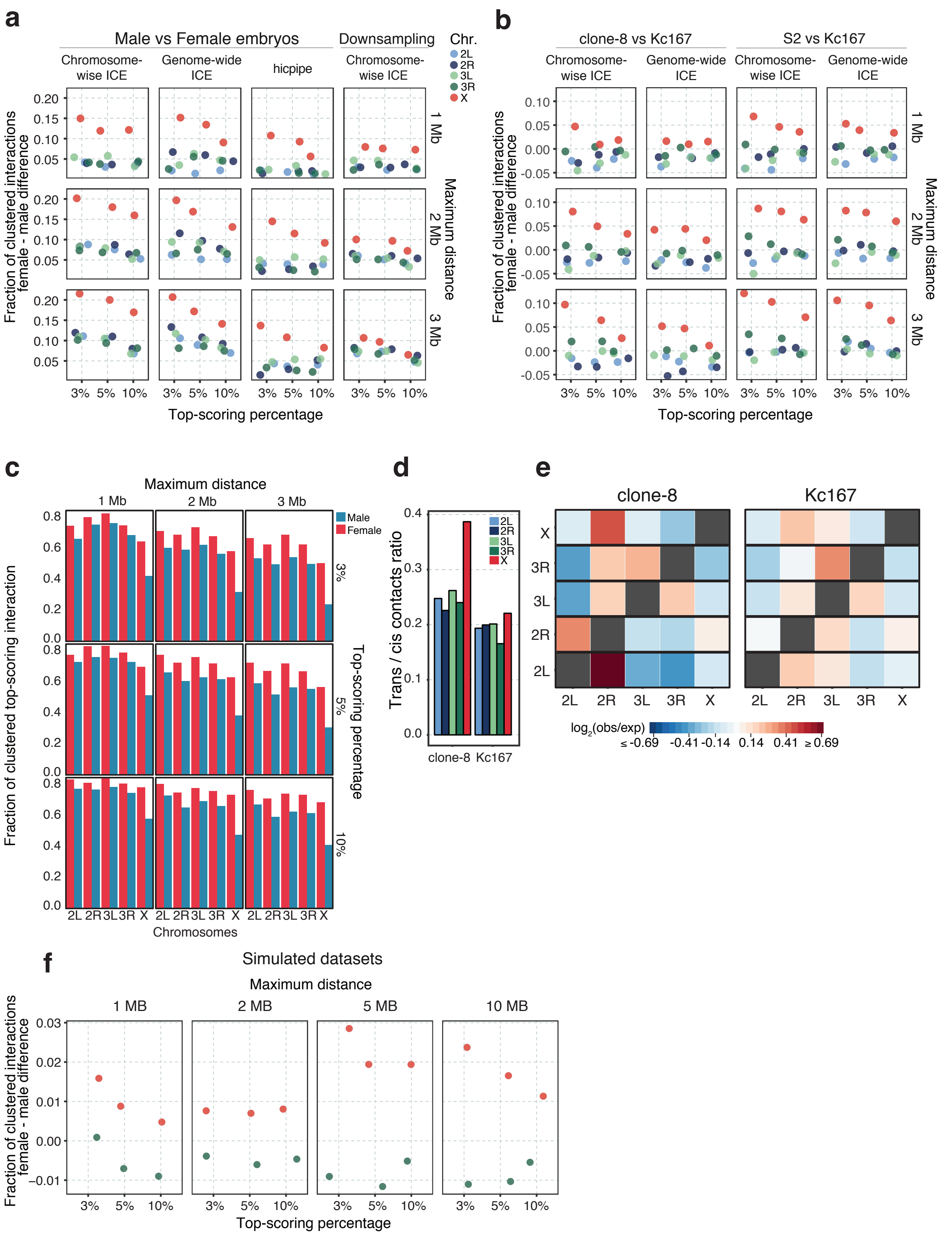

Supplementary Figure 3 - ChrX specific patterns of cis and trans interactions are consistent across variations of analysis parameters. (a) Difference in the fraction of clustered top scoring interactions (female – male, y-axis) is shown for each autosome arm and chrX (see colour legend). Top scoring interactions were detected in 25Kb bins Hi-C data for sex-sorted embryos processed with three different normalization procedures

(chromosome-wise ICE, genome-wide ICE, hicpipe). The analysis was also repeated on down-sampled female Hi-C data (reads from each cis chromosomal Hi-C map was down-sampled to the same coverage of the corresponding male chromosome) then normalized by chromosome-wise ICE. Top-scoring interactions were selected at various percentile thresholds: top scoring interactions percentage (top 3%, 5% and 10%) as indicated on x-axis. Clustered top scoring interactions were defined as described in Methods section, by aggregating neighbouring data points selected as top scoring. The fraction of clustered data points is defined considering distances up to 1Mb (upper row), 2Mb (middle row) or 3Mb (bottom row), as indicated on the side. **(b)** The same analysis as in panel **a** was repeated on independent Hi-C datasets for male (clone-8, S2) and female (Kc167) cell lines. Two alternative normalizations (chromosome-wise ICE and genome-wide ICE) were considered, as well as multiple thresholds on top scoring interaction quantiles (x-axis) and maximum distance between interactions: up to 1Mb (upper row), 2Mb (middle row) or 3Mb (bottom row), as indicated on the side. **(c)** Proportion of clustered top scoring interactions (y-axis) related to panel **a** (chromosome-wise ICE normalization) are shown separately for male (blue) and female (red) samples across each chromosome (x-axis). Multiple thresholds on top scoring interactions quantiles were considered (top 3%, 5% and 10%, as indicated on the right side for each row), as well as maximum distance between interactions up to 1Mb (left column), 2Mb (central column) or 3Mb (right column). **(d)** The relative amount of trans interaction for each chromosome is shown as the ratio of the number of *trans-* over *cis*-mapping read pairs for each chromosome in male (clone-8) and female (Kc167) cell lines Hi-C datasets. **(e)** The propensity of each chromosome to participate in trans interactions is shown for male (clone-8 - left) and female (Kc167 - right) cell lines. The trans-mapping read pairs originating from each chromosome (rows) are divided based on the target chromosome (columns) and their number compared to the random expectation. In the random model, trans reads originating from any chromosome are expected to be uniformly distributed over the target chromosomes, after adjusting for their length and copy number (see Methods). The $\log_2$ ratio between the observed and expected fraction of trans read counts is reported as a color gradient. Note that the heatmap is not expected to be symmetrical because the expected number of interactions is different depending on the origin vs target chromosome pairs. The diagonal is grey as cis-interactions not considered. **(f)** The same analysis as in panel a and b is reported for the polymer folding simulations. The difference in proportion of clustered data points for chr3R is reported considering paired chr3R in the presence of chrX with or without pairing. On the other hand, for chrX the difference is reported between chrX with or without pairing. Similar to panel a and b, multiple thresholds of top scoring interaction quantiles (x-axis) and multiple maximum distance measures (top; 1MB, 2MB, 5MB, 10MB) were adopted.

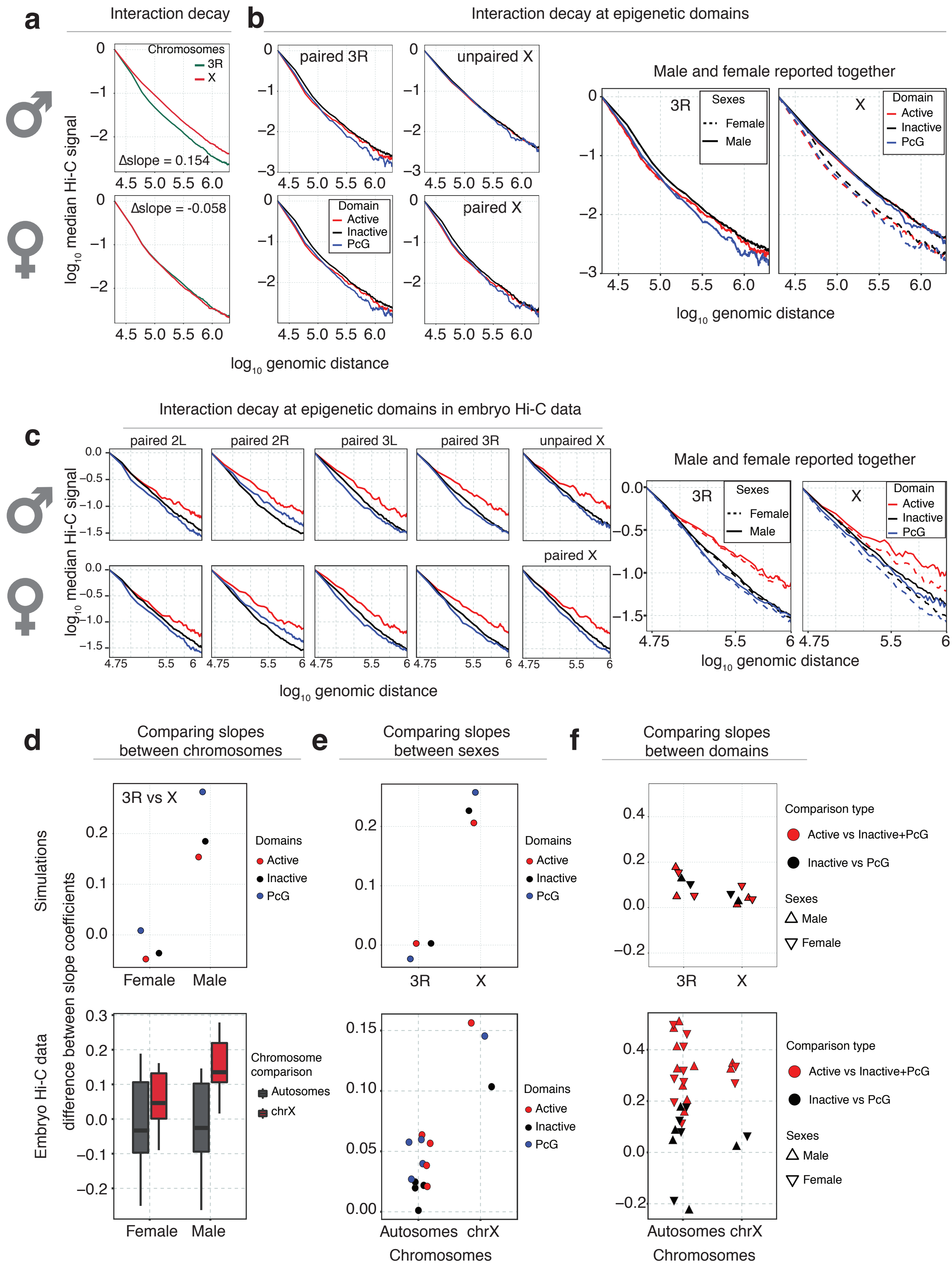

Supplementary Figure 4 - chrX specific changes in Hi-C interaction decay are partly associated to the absence of pairing on chrX (a) Interaction signal decay with distance is shown in a log-log plot for Hi-C data obtained using polymer folding simulations for chr3R and chrX in male (top, paired 3R & unpaired X) and female (bottom, paired 3R & paired X). For any given genomic distance (log_{10} bp distance on x-axis) the median log_{10}

Hi-C signal binned at 20Kb is reported (y-axis). Distances ranging from 20Kb (1 bin distance in a 20Kb binned matrix) to 2Mb are shown. The difference (Δslope) of the rate of Hi-C signal decay (slope coefficient) is reported. **(b)** Interaction signal decay with distance is shown in a log-log plot for each epigenetic class as predicted by polymer folding simulations for paired chr3R (top left) in the presence of an unpaired chrX (top right) and paired chr3R (bottom left) in the presence of a paired chrX (bottom right). For each genomic bin, the interaction decay frequency plot is classified based on the epigenetic class of its chromatin domain. For any given genomic distance ($\log_{10}$ bp distance on x-axis) the median $\log_{10}$ Hi-C signal binned at 20Kb is reported (y-axis). Distances ranging from 20Kb to 2Mb are shown. On the right, the chromosomes from the two simulations are reported together to highlight observable differences between the paired and unpaired chrX. No significant differences are observed between the two paired states of chr3R in the presence or absence of pairing in chrX. **(c)** Interaction signal decay with distance is shown for each epigenetic state in a log-log plot for embryo Hi-C data binned at 25Kb and normalised with genome-wide ICE. For each genomic bin, the interaction decay frequency plot is classified based on the epigenetic class of its chromatin domain. For any given genomic distance ($\log_{10}$ bp distance on x-axis) the median $\log_{10}$ Hi-C signal is reported (y-axis) for each epigenetic state. Similar to panel b, chr3R and chrX is reported together for both sexes to highlight observable differences between the sexes. **(d)** The slope coefficients were computed for each epigenetic state interaction decay considering distances <400Kb (≤5.6 in $\log_{10}$ distance) and the pairwise difference of slope coefficients between chromosomes (y-axis) is reported for the sexes (x-axis) in the polymer simulations (top, Female - paired X vs paired 3R; Male - unpaired X vs paired 3R) and embryo Hi-C data (bottom). In the boxplot the median is marked as horizontal line, the boxes mark the interquartile range (IQR), the whiskers extend up to 1.5 IQR. **(e)** The slope coefficients were computed for each epigenetic state interaction decay considering distances <400Kb (≤5.6 in $\log_{10}$ distance) and the pairwise difference of slope coefficients between sexes (y-axis) is reported for autosomes (chr3R in simulated Hi-C data) and chrX (x-axis). **(f)** The slope coefficients were computed for each epigenetic state interaction decay considering distances <400Kb (≤5.6 in $\log_{10}$ distance) and the pairwise difference of slope coefficients between epigenetic states (y-axis) is reported for autosomes (chr3R in simulated Hi-C data) and chrX (x-axis).

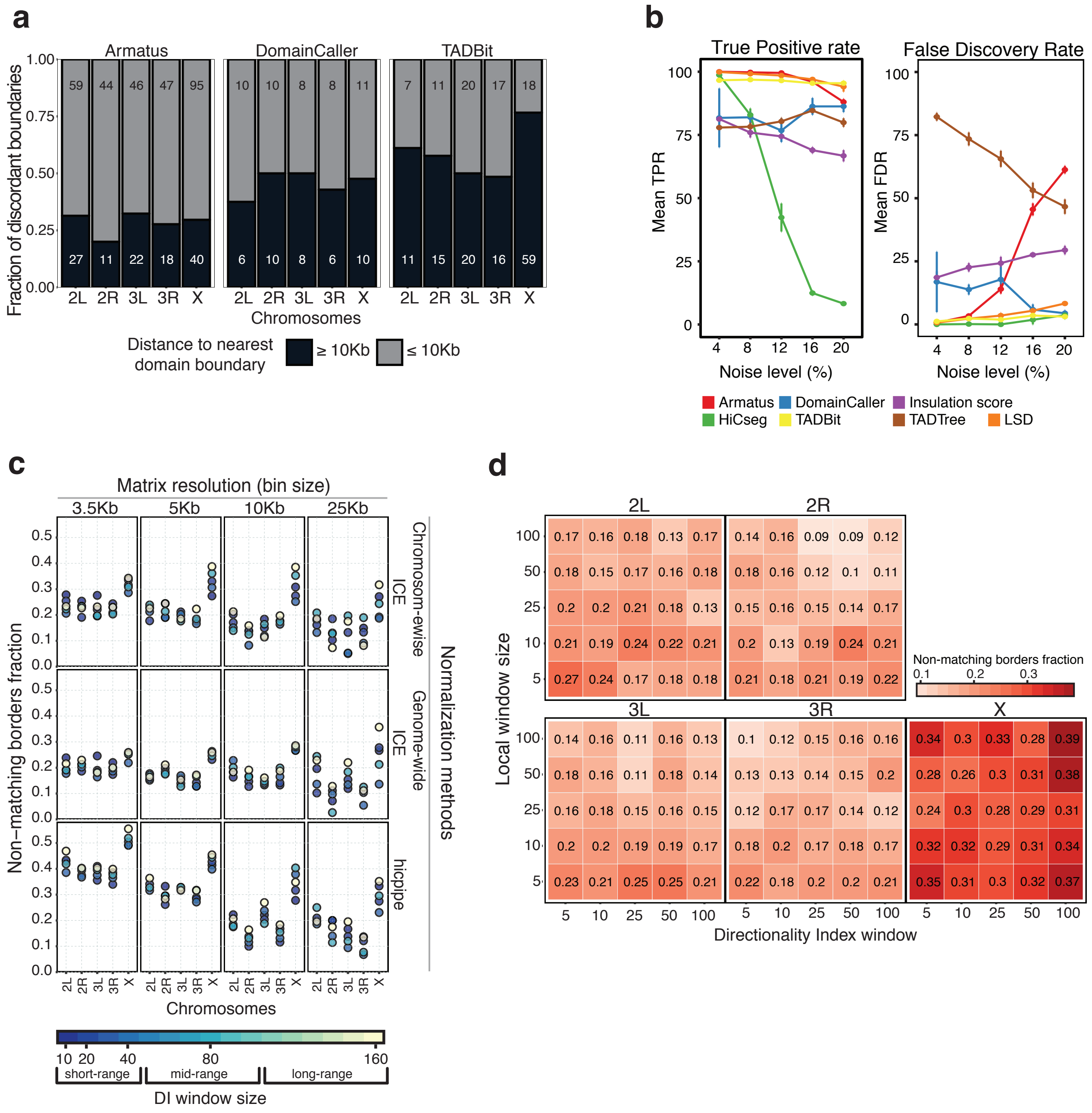

Supplementary Figure 5 - ChrX specific domain borders differences are robust and independent of analysis parameters. (a) The stacked barplot shows the fraction of non-matching TAD boundaries in male grouped by distance to the nearest TAD border in the female counterpart as identified on 10Kb binned Hi-C matrices three alternative published algorithms: Armatus, DomainCaller and TADbit (as indicated) calling procedures. The proportion of non-matching boundaries (y-axis) which are just one bin apart are shown in light grey in the stacked barplot (i.e. ≤10Kb - see colour legend), those at larger distances are in black. The numbers reported within the stacked bars indicate the count of non-matching boundaries for each category. **(b)** The Local score differentiator (LSD) domain border calling procedure was benchmarked against previously proposed TAD calling procedures on simulated Hi-C matrices (see Methods). The true positive rate (TPR, left plot) and the false discovery rate (FDR, right plot) are reported across simulated datasets with increasing level of noise (x-axis). Error bars are mean±standard error of the mean (SEM) over n=5 simulated datasets. **(c)** Fraction of non-matching domain borders (y-axis)

in male vs female samples is reported for each chromosomes (x-axis) based on LSD analysis of Hi-C data normalized with three alternative procedures: chromosome-wise ICE (upper row), genome-wide ICE (middle row) and hicpipe (bottom row), as indicated on the right axes. Alternative resolution in the matrices (bins size 3.5Kb, 5Kb, 10Kb or 256Kb as indicated on the top), as well as variations in LSD parameters including the directionality index window size (n - color scale legend) and the local window size (m), which is set to 2 times the DI window size for this analysis. ChrX consistently shows larger differences. **(d)** Fraction of non-matching domain borders identified with LSD analysis on male vs female 10Kb binned Hi-C data normalized by chromosome-wise ICE after down-sampling of each female chromosome sequencing reads to match the coverage of the male counterpart. The proportion of non-matching domain boundaries between the male and down-sampled female samples is reported (color scale and numbers in the grid) for each chromosome (upper axis labels) across various combinations of analysis parameters including local window size (m - y-axis) and DI window size (n - x-axis).

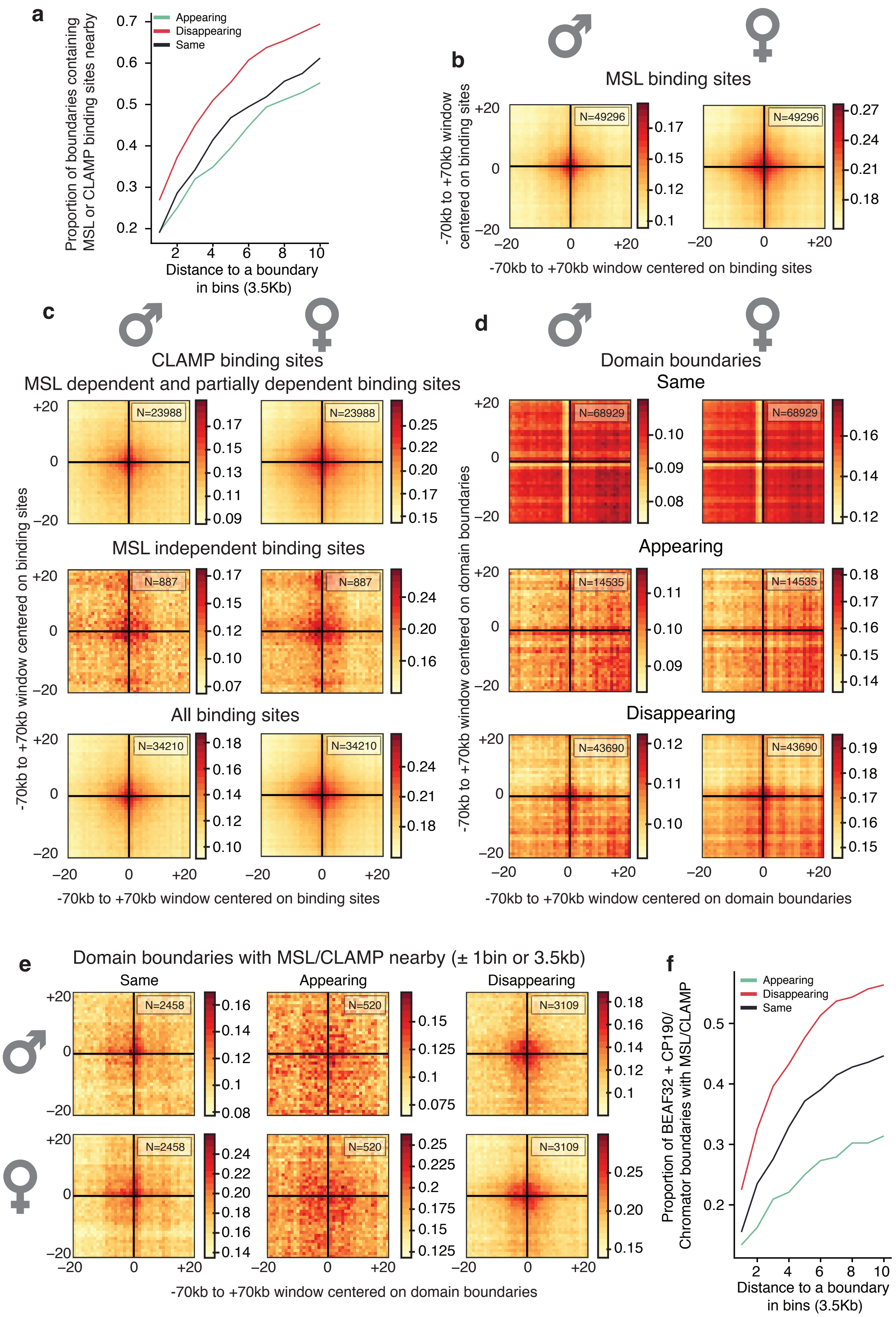
a
Appearing
Disappearing
Same
Proportion of boundaries containing MSL or CLAMP binding sites nearby
Distance to a boundary in bins (3.5Kb)
b
MSL binding sites
N=49296
-70kb to +70kb window centered on binding sites
c
CLAMP binding sites
MSL dependent and partially dependent binding sites
N=23988
MSL independent binding sites
N=887
All binding sites
N=34210
-70kb to +70kb window centered on binding sites
d
Domain boundaries
Same
N=68929
Appearing
N=14535
Disappearing
N=43690
-70kb to +70kb window centered on domain boundaries
e
Domain boundaries with MSL/CLAMP nearby (± 1bin or 3.5kb)
Same
Appearing
Disappearing
N=2458
N=520
N=3109
-70kb to +70kb window centered on domain boundaries
f
Appearing
Disappearing
Same
Proportion of BEAF32 + CP190/ Chromator boundaries with MSL/CLAMP
Distance to a boundary in bins (3.5Kb)

Supplementary Figure 6 - ChrX specific domain borders harbouring nearby MSL or CLAMP binding sites participate in long-range interactions. (a) Distance relationship between MSL or CLAMP binding sites and domain boundary classes is shown. Considering each class of domain boundaries (Disappearing - Red, Appearing - Green and Same - Black), the proportion of boundaries containing a MSL or CLAMP binding site (y-axis) nearby is plotted against the distance used to classify a MSL or CLAMP binding site as being near to a boundary belonging to a particular class (x-axis). Distance to a nearby MSL or CLAMP binding site ranges from 1 bin (±3.5Kb) to 10 bins (±35Kb). **(b)** Average pairwise interaction plots are shown for all MSL binding site definitions (Supplementary Data 3, see Methods) in male (left) and female (right) embryo Hi-C data binned at 3.5Kb and normalised using chromosome-wise ICE. 70Kb non-overlapping windows (140Kb considering both upstream and downstream) were considered around each pair of MSL binding sites. **(c)** Average pairwise interaction plots are shown for CLAMP binding sites in male (left) and female (right) embryo Hi-C data binned at 3.5Kb and normalised with chromosome-wise ICE. The CLAMP binding sites are stratified by MSL binding dependency (Top - Completely or partially dependent on MSL binding; Middle - MSL binding independent; Bottom - All CLAMP binding sites). 70Kb non-overlapping windows (140Kb considering both upstream and downstream) were considered around each pair of CLAMP binding sites. **(d)** Average pairwise interaction plots are shown for domain boundary classes (Same, Appearing, Disappearing) in male (left) and female (right) embryo Hi-C data binned at 3.5Kb and normalised with chromosome-wise ICE. 70Kb non-overlapping windows (140Kb considering both upstream and downstream) were considered around each pair of domain boundaries. **(e)** Average pairwise interaction plots are shown for domain boundary classes (left - Same, middle - Appearing, right - Disappearing) which are near to a MSL or CLAMP binding site in male (top) and female (bottom) embryo Hi-C data binned at 3.5Kb and normalised using chromosome-wise ICE. **(f)** Distance relationship between MSL or CLAMP binding sites and domain boundaries containing CP190 or Chromator peaks in conjunction with BEAF-32 is shown. Considering each class of domain boundaries (Disappearing - Red, Appearing - Green and Same - Black) harbouring a CP190 or Chromator peak in conjunction with BEAF-32, the proportion of such boundaries containing a MSL or CLAMP binding site (y-axis) nearby is plotted. The distance used to classify a MSL or CLAMP binding site as being near to a boundary belonging to a particular class (x-axis) ranges from 1 bin (±3.5Kb) to 10 bins (±35Kb).

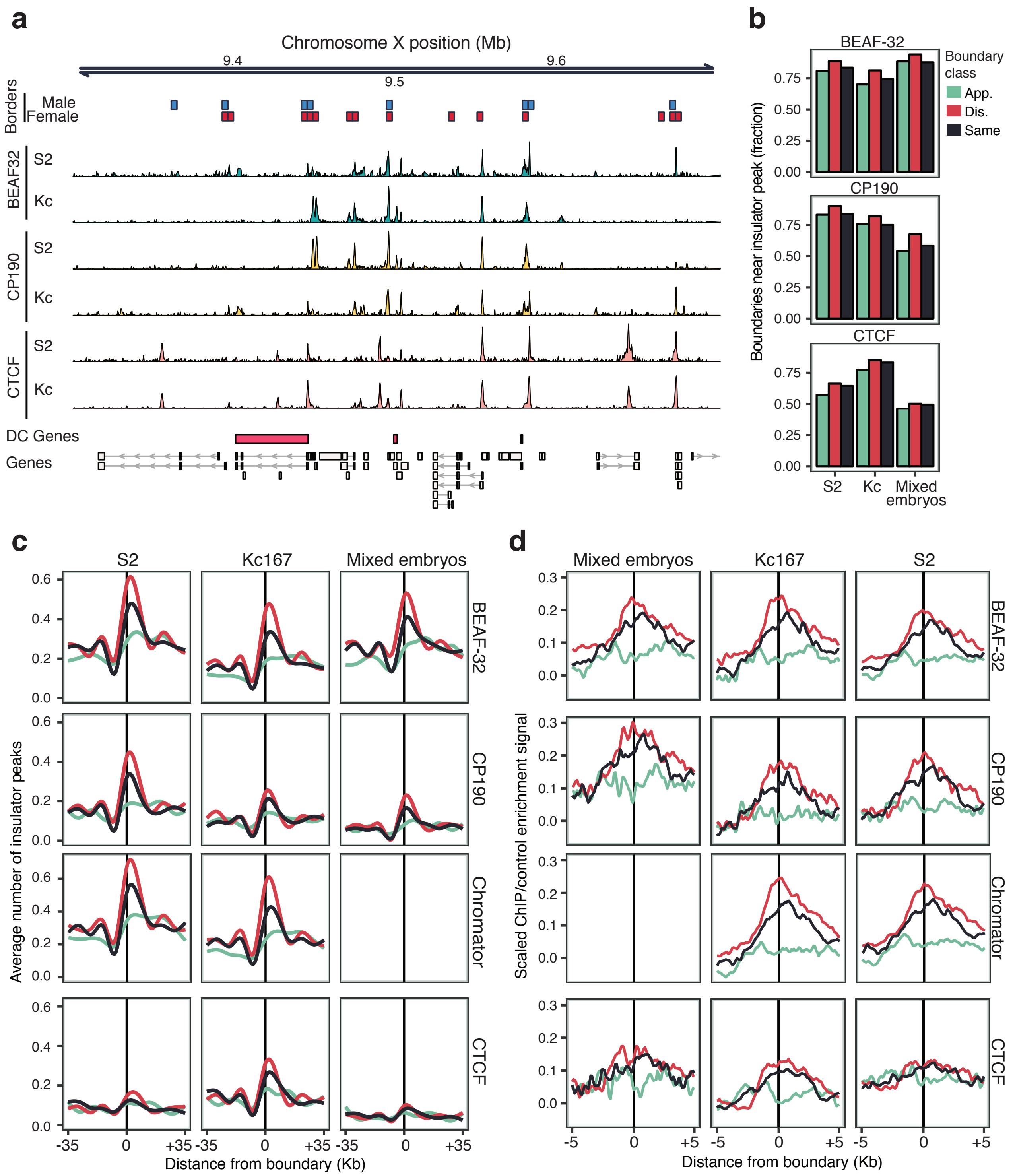

Supplementary Figure 7 - Chromatin insulators binding around changing domain boundaries. (a) Representative region of chrX with genomic tracks for binding profiles of insulators expected to be located at domain boundaries (BEAF32, CP190 and CTCF) alongside male (blue boxes) and female (red boxes) domain boundaries identified in sex-sorted embryos Hi-C matrices binned at 3.5Kb. Insulator binding profiles are from male (S2) and female (Kc167) cell lines. Genes responsive to dosage compensation are also marked in the bottom track (magenta boxes) along with gene annotations for exon/intron structure and sense of transcription. **(b)** Frequency of domain boundaries (classes as defined in sex-sorted male and female embryos) near an insulator binding peak: in male

(S2), female (Kc167) or mixed embryos ChIP-chip data. The fraction of domain boundaries with of each class with an insulator binding peak within 35Kb (10 Hi-C bins distance) is reported. **(c)** The distribution of insulators (BEAF32, CP190, CTCF) binding peaks around domain boundaries stratified by classes are show considering a 35Kb (10 bins distance). The average number of peaks are shown for S2, Kc167, and mixed embryos (see Methods). **(d)** The average of insulators ChIP-chip enrichment signal from the modENCODE data files is reported. modENCODE enrichment is lowess smoothed (500bp bandwidth) M values (log_2 signal intensities of CHiP over control). Enrichment values are scaled to the 99th as described in Methods. The average signal of insulators binding (BEAF32, CP190 and CTCF) is shown around the domain boundaries of each class.

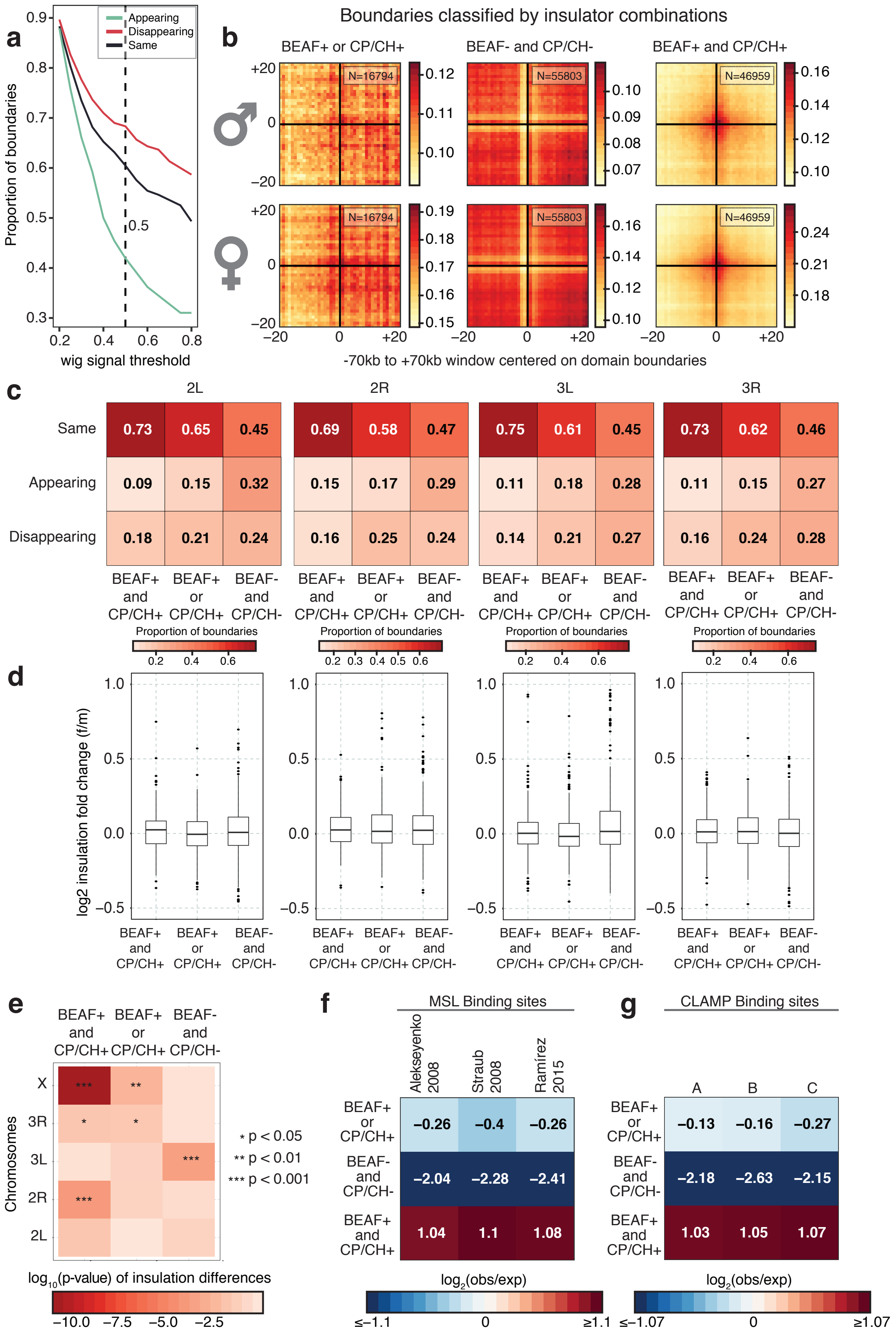

	BEAF+ and CP/CH+ (2L)	BEAF+ or CP/CH+ (2L)	BEAF- and CP/CH- (2L)	BEAF+ and CP/CH+ (2R)	BEAF+ or CP/CH+ (2R)	BEAF- and CP/CH- (2R)	BEAF+ and CP/CH+ (3L)	BEAF+ or CP/CH+ (3L)	BEAF- and CP/CH- (3L)	BEAF+ and CP/CH+ (3R)	BEAF+ or CP/CH+ (3R)	BEAF- and CP/CH- (3R)
Same	0.73	0.65	0.45	0.69	0.58	0.47	0.75	0.61	0.45	0.73	0.62	0.46
Appearing	0.09	0.15	0.32	0.15	0.17	0.29	0.11	0.18	0.28	0.11	0.15	0.27
Disappearing	0.18	0.21	0.24	0.16	0.25	0.24	0.14	0.21	0.27	0.16	0.24	0.28

	Alekseyenko 2008	Straub 2008	Ramírez 2015
BEAF+ or CP/CH+	−0.26	−0.4	−0.26
BEAF- and CP/CH-	−2.04	−2.28	−2.41
BEAF+ and CP/CH+	1.04	1.1	1.08

	A	B	C
BEAF+ or CP/CH+	−0.13	−0.16	−0.27
BEAF- and CP/CH-	−2.18	−2.63	−2.15
BEAF+ and CP/CH+	1.03	1.05	1.07

Supplementary Figure 8 - chrX specific domain boundaries containing CP190 or Chromator in conjunction with BEAF-32 are associated to MSL or CLAMP binding sites. (a) In each boundary class (Same, Appearing, Disappearing), we plot the proportion of boundaries (y-axis) containing enrichment signal above a certain threshold for any one insulator protein (BEAF32, CP190, Chromator) in one of either male (S2) or female (Kc167) datasets from modENCODE. The threshold used to classify if a protein has high signal ranges between 0.2 and 0.8 (x-axis). The threshold used in the study (black vertical line) is 0.5. **(b)** Average pairwise interaction plots are shown for domain boundaries after classification on the basis of combinatorial binding of insulator proteins in male (top) and female (bottom) embryo Hi-C data binned at 3.5Kb and normalised with chromosome-wise ICE. BEAF+ or CP/CH+ boundaries have high enrichment signal for BEAF-32 or one of either CP190 or Chromator, BEAF- and CP/CH- boundaries have low signal for all three insulator proteins, and BEAF+ and CP/CH+ boundaries have high signal for BEAF-32 and one of either CP190 or Chromator. 70Kb non-overlapping windows (140Kb considering both upstream and downstream) were considered around each pair of domain boundaries. **(c)** The proportion of boundary classes (y-axis - Same, Appearing, Disappearing) represented in each of the boundary classes after reclassification on the basis of insulator binding (x-axis) is shown for autosomes (top). All columns sum to 1, the colour gradient is mapped to the proportion indicated in each cell. In general, Same boundaries represent the highest proportion of boundaries showcasing high binding signal for CP190 or Chromator in conjunction with BEAF-32. **(d)** log_2 insulation ratio (y-axis; male/female) is shown for boundary classes defined on the basis of combinatorial binding of insulators in the autosomes. For each boxplot the median is marked as horizontal line, the boxes mark the interquartile range (IQR), the whiskers extend up to 1.5 IQR and individual data points are shown for outliers beyond this range. **(e)** Wilcoxon p-values are plotted for pairwise wilcoxon test on male and female insulation scores at boundaries classified on the basis of combinatorial binding of insulators. Heatmap colour is mapped to the log_{10} p-values and the significance level is also indicated. **(f)** Enrichment (log_2 observed over expected ratio) of MSL binding sites around the domain borders grouped by combinatorial binding of insulators is shown. Three alternative definitions of MSL binding sites from three different laboratories were considered: Kuroda[4], Becker[5] and Akhtar[6] laboratories. The expected frequency was computed based on random uniform distribution of MSL binding sites along chrX. **(g)** Enrichment (log_2 observed over expected ratio) of CLAMP binding sites around the domain borders grouped by combinatorial binding of insulators is shown. Three groups of CLAMP binding sites as defined in Soruco et al.[7] are considered: MSL-dependent binding of CLAMP (A), Partially dependent on MSL binding (B) and MSL independent binding of CLAMP (C). The expected frequency was computed based on random uniform distribution of CLAMP binding sites along chrX.

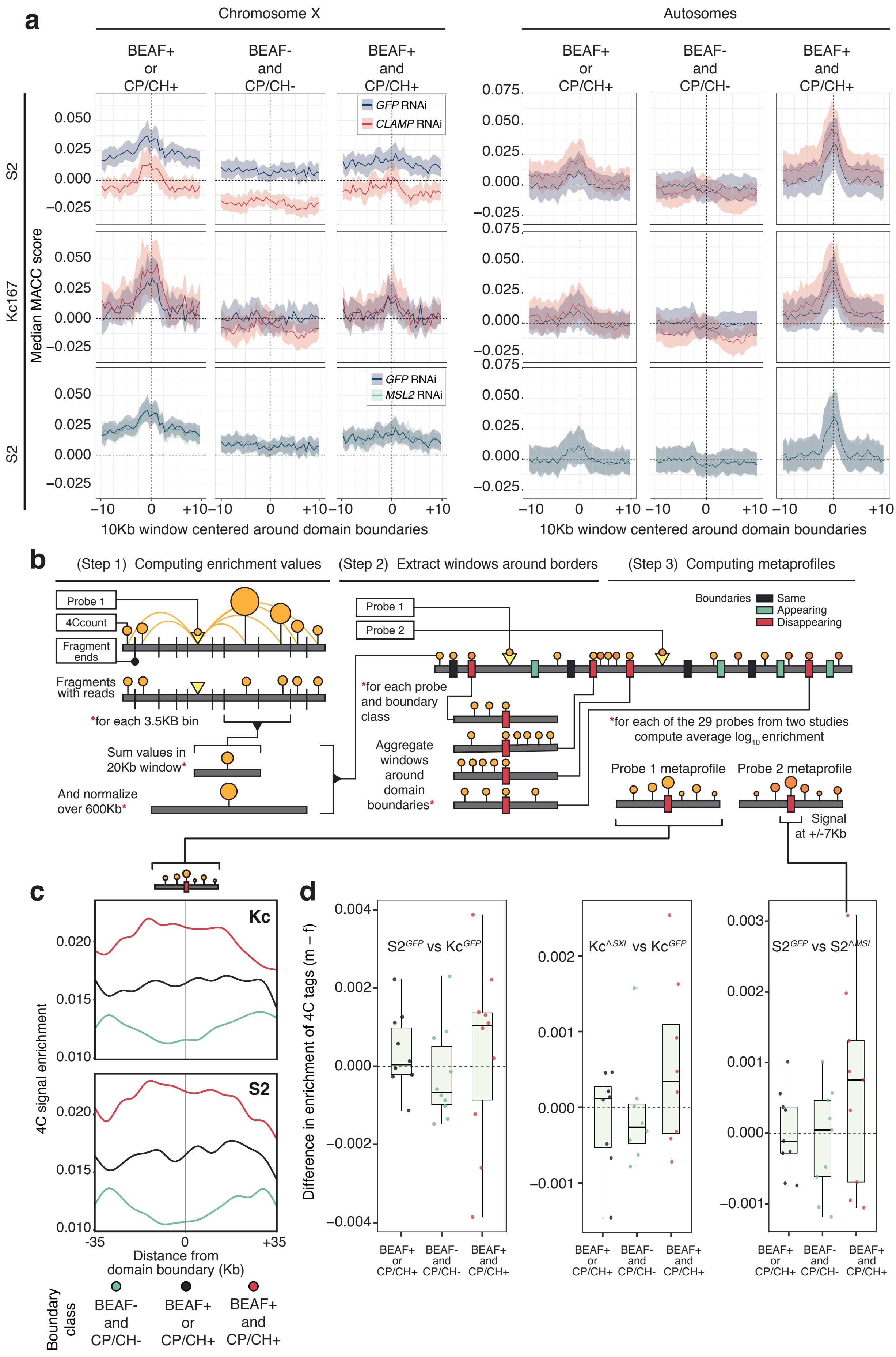
a
Chromosome X
Autosomes
BEAF+ or CP/CH+
BEAF- and CP/CH-
BEAF+ and CP/CH+
GFP RNAi
CLAMP RNAi
MSL2 RNAi
S2
Kc167
S2
Median MACC score
10Kb window centered around domain boundaries
b
(Step 1) Computing enrichment values
(Step 2) Extract windows around borders
(Step 3) Computing metaprofiles
Probe 1
4Ccount
Fragment ends
Fragments with reads
*for each 3.5KB bin
Sum values in 20Kb window*
And normalize over 600Kb*
Probe 1
Probe 2
Boundaries
Same
Appearing
Disappearing
*for each probe and boundary class
Aggregate windows around domain boundaries*
*for each of the 29 probes from two studies compute average log10 enrichment
Probe 1 metaprofile
Probe 2 metaprofile
Signal at +/-7Kb
c
Kc
S2
4C signal enrichment
Distance from domain boundary (Kb)
Boundary class
BEAF- and CP/CH-
BEAF+ or CP/CH+
BEAF+ and CP/CH+
d
Difference in enrichment of 4C tags (m – f)
S2GFP vs KcGFP
KcΔSXL vs KcGFP
S2GFP vs S2ΔMSL
BEAF+ or CP/CH+
BEAF- and CP/CH-
BEAF+ and CP/CH+

Supplementary Figure 9 - Accessibility at chrX specific domain boundaries is affected by CLAMP knockdown and show differential pattern in 4C meta-profiles. (a) MNase-seq metaprofiles for BEAF+ and CP/CH+ are shown in datasets obtained from Urban et al.[8]. Metaprofiles are separated into chromosome X (left) and autosomes (right). both S2 (male, top and bottom) and Kc167 (female, middle) is shown labelled by the condition: GFP RNAi (black), CLAMP RNAi (red) and MSL2 RNAi (green) (40th to 60th percentile interval is shown as a shaded area). CLAMP RNAi MACC score for S2 cells is noticeably lower in chromosome X, but the same is not observed in autosomes or Kc167 cell lines. **(b)** Schematic workflow of 4C data aggregation. First, 4C read counts per restriction fragment are transformed into enrichment values as in Ramirez et al.[6]. Namely, the number of fragments with reads is counted over 20Kb windows and compared against the expected background computed over larger (600Kb windows). An enrichment signal computed with this procedure is assigned to each 3.5Kb bin of the chrX. Then, for each 4C profile originated from each 4C probe, the enrichment signal around each domain boundary, grouped by insulator classes, is extracted and the average $\log_{10}$ enrichment profile is computed (meta-profile), as shown in panel **(c)** For summarizing the signal enrichment around domain boundaries across multiple probes, the central part of the metaprofiles (mean signal in +/-7Kb window around domain boundaries) is considered as individual data points in the boxplots as in panel d. **(d)** Difference of mean 4C-seq enrichment signal (±7Kb) for each class of domain boundaries based on metaprofiles for each 4C probe (each data point in the plot) from Schauer et al.[9] in male (S2) and female (Kc) cell lines. For each box the median is marked as horizontal line, the boxes mark the interquartile range (IQR), the whiskers extend up to 1.5 IQR and all individual data points are shown.

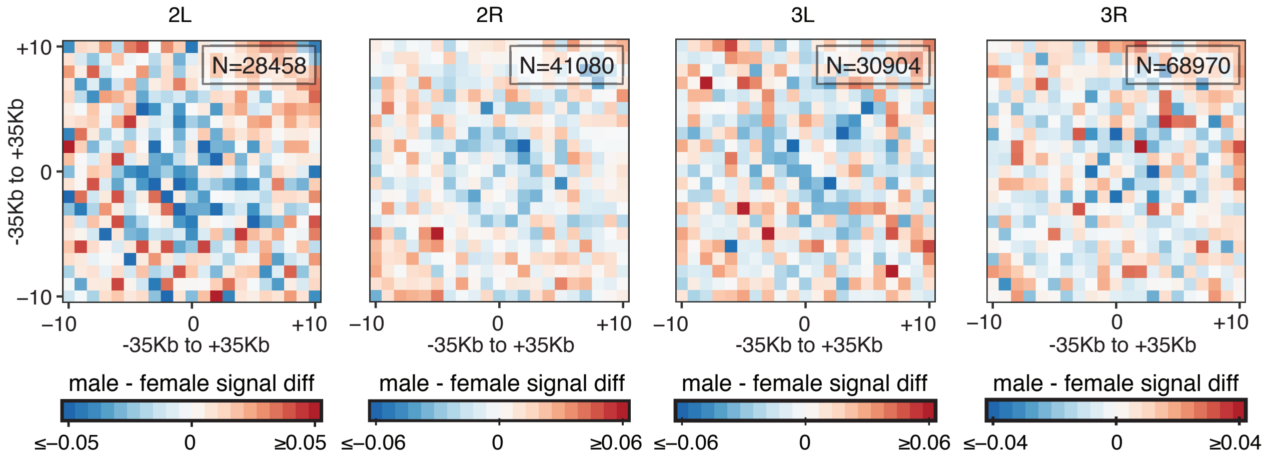

Supplementary Figure 10 - BEAF+ and CP/CH+ boundaries do not showcase any noticeable difference in pairwise interaction profiles. Considering BEAF+ and CP/CH+ boundaries near active genes, average local background normalized interaction profiles were created in 3.5KB binned male and female Hi-C maps for all non-overlapping pairwise combinations of boundaries using a window of 35KB (10 bins in 3.5KB Hi-C maps) around boundaries and the difference (male - female) between the two average interaction profiles is shown for autosomes. The autosomes do not showcase a consistent observable difference neat the centre where BEAF+ and CP/CH+ boundaries interact.

Supplementary References

1 Yaffe, E. & Tanay, A. Probabilistic modeling of Hi-C contact maps eliminates systematic biases to characterize global chromosomal architecture. *Nature genetics* **43**, 1059-1065, doi:10.1038/ng.947 (2011).

2 Imakaev, M. *et al.* Iterative correction of Hi-C data reveals hallmarks of chromosome organization. *Nature methods* **9**, 999-1003, doi:10.1038/nmeth.2148 (2012).

3 Giorgetti, L. *et al.* Predictive polymer modeling reveals coupled fluctuations in chromosome conformation and transcription. *Cell* **157**, 950-963, doi:10.1016/j.cell.2014.03.025 (2014).

4 Alekseyenko, A. A. *et al.* A sequence motif within chromatin entry sites directs MSL establishment on the Drosophila X chromosome. *Cell* **134**, 599-609, doi:10.1016/j.cell.2008.06.033 (2008).

5 Straub, T., Grimaud, C., Gilfillan, G. D., Mitterweger, A. & Becker, P. B. The chromosomal high-affinity binding sites for the Drosophila dosage compensation complex. *PLoS Genet* **4**, e1000302, doi:10.1371/journal.pgen.1000302 (2008).

6 Ramirez, F. *et al.* High-Affinity Sites Form an Interaction Network to Facilitate Spreading of the MSL Complex across the X Chromosome in Drosophila. *Molecular cell* **60**, 146-162, doi:10.1016/j.molcel.2015.08.024 (2015).

7 Soruco, M. M. *et al.* The CLAMP protein links the MSL complex to the X chromosome during Drosophila dosage compensation. *Genes & development* **27**, 1551-1556, doi:10.1101/gad.214585.113 (2013).

8 Urban, J. *et al.* Enhanced chromatin accessibility of the dosage compensated Drosophila male X-chromosome requires the CLAMP zinc finger protein. *PLoS One* **12**, e0186855, doi:10.1371/journal.pone.0186855 (2017).

9 Schauer, T. *et al.* Chromosome topology guides the Drosophila Dosage Compensation Complex for target gene activation. *EMBO Rep*, doi:10.15252/embr.201744292 (2017).
