# Supplementary material for: Global chromatin conformation differences in the Drosophila dosage compensated chromosome X
Source: Nat Commun. 2019 Nov 25;10:5355. doi: 10.1038/s41467-019-13350-8 (PMC6877619; doi:10.1038/s41467-019-13350-8)
Supplement: Supplementary file 3 — Description of Additional Supplementary Files [file 41467_2019_13350_MOESM3_ESM.pdf]

Description of Additional Supplementary Files

File Name: Supplementary Data 1
Description: Read counts for each chromosome pair and male over female ratios of cis contacts.

File Name: Supplementary Data 2
Description: Domain boundaries as predicted by Local Score Differentiator (LSD) on sex-sorted male and female embryos Hi-C matrices binned at 3.5Kb. The boundaries have been grouped by their change classes (Same, Appearing, Disappearing) and their insulator combination classes.

File Name: Supplementary Data 3
Description: MSL binding site mid-points as defined by three alternative groups (column 4).

File Name: Supplementary Data 4
Description: CLAMP binding site mid-points grouped by their MSL binding specificity (Group A - MSL dependent binding, Group B - Partial dependence on MSL binding, Group C - Independent of MSL binding).

File Name: Supplementary Data 5
Description: 4C-seq probes from Schauer et al. and Ramiréz et al. with their corresponding GEO IDs. Only the used GEO IDs are listed here. 4C seq probes originating from each study are listed in separated sheets.

File Name: Supplementary Data 6
Description: Gene expression values for each gene as obtained from Zhang et al. Genes have been grouped by their expression status in autosomes and dosage compensation status in chromosome X.

File Name: Supplementary Data 7
Description: Core-set of chrX domain boundaries.
