# Supplementary material for: Global chromatin conformation differences in the Drosophila dosage compensated chromosome X
Source: Nat Commun. 2019 Nov 25;10:5355. doi: 10.1038/s41467-019-13350-8 (PMC6877619; doi:10.1038/s41467-019-13350-8)
Supplement: Supplementary file 11 — Reporting Summary [file 41467_2019_13350_MOESM11_ESM.pdf]

nature research

Corresponding author(s): Francesco Ferrari

Last updated by author(s): Oct 8, 2019

Reporting Summary

Nature Research wishes to improve the reproducibility of the work that we publish. This form provides structure for consistency and transparency in reporting. For further information on Nature Research policies, see Authors & Referees and the Editorial Policy Checklist.

Statistics

For all statistical analyses, confirm that the following items are present in the figure legend, table legend, main text, or Methods section.

n/a	Confirmed	
☐	☒	The exact sample size (*n*) for each experimental group/condition, given as a discrete number and unit of measurement
☐	☒	A statement on whether measurements were taken from distinct samples or whether the same sample was measured repeatedly
☐	☒	The statistical test(s) used AND whether they are one- or two-sided<br>*Only common tests should be described solely by name; describe more complex techniques in the Methods section.*
☐	☒	A description of all covariates tested
☐	☒	A description of any assumptions or corrections, such as tests of normality and adjustment for multiple comparisons
☐	☒	A full description of the statistical parameters including central tendency (e.g. means) or other basic estimates (e.g. regression coefficient) AND variation (e.g. standard deviation) or associated estimates of uncertainty (e.g. confidence intervals)
☐	☒	For null hypothesis testing, the test statistic (e.g. *F*, *t*, *r*) with confidence intervals, effect sizes, degrees of freedom and *P* value noted<br>*Give P values as exact values whenever suitable.*
☒	☐	For Bayesian analysis, information on the choice of priors and Markov chain Monte Carlo settings
☒	☐	For hierarchical and complex designs, identification of the appropriate level for tests and full reporting of outcomes
☐	☒	Estimates of effect sizes (e.g. Cohen's *d*, Pearson's *r*), indicating how they were calculated

Our web collection on statistics for biologists contains articles on many of the points above.

Software and code

Policy information about availability of computer code

Data collection	Not applicable
Data analysis	As detailed in methods section: hiclib (2016-07-14 version - commit fe3817a); cooler (v0.3.0); hicpipe (v1.03); bowtie (v1.1.2) and version 2.2.9 for specific analyses as indicated; Armatus (v2.1) ; DomainCaller; TADBit (v0.1_alpha.360); deepTools (version 2.5.3); source for LSD analysis is available in the public respository https://bitbucket.org/koustavpal1988/fly_dc_structuralchanges_2018/

For manuscripts utilizing custom algorithms or software that are central to the research but not yet described in published literature, software must be made available to editors/reviewers. We strongly encourage code deposition in a community repository (e.g. GitHub). See the Nature Research guidelines for submitting code & software for further information.

Data

Policy information about availability of data

All manuscripts must include a data availability statement. This statement should provide the following information, where applicable:

- Accession codes, unique identifiers, or web links for publicly available datasets
- A list of figures that have associated raw data
- A description of any restrictions on data availability

All genomics data used in the manuscript have been previously released in public repositories as indicated in the manuscript or methods details above. These include GEO datasets for Hi-C (GSE94115), MNAse-seq (GSE99893), ATAC-seq (GSE101581), RNA-seq (GSE16344), ChIP-seq (GSE39271) and modENCODE datasets for ChIP-chip (IDs 3745, 3748, 277, 908, 274, 925, 279, 3281, 5130, 5131, 5057, as detailed in the methods section) and DNase-seq (IDs 3324 and 3325).

Field-specific reporting

Please select the one below that is the best fit for your research. If you are not sure, read the appropriate sections before making your selection.

☒ Life sciences ☐ Behavioural & social sciences ☐ Ecological, evolutionary & environmental sciences

For a reference copy of the document with all sections, see nature.com/documents/nr-reporting-summary-flat.pdf

Life sciences study design

All studies must disclose on these points even when the disclosure is negative.

Sample size	The manuscript is using previously released genomics datasets. The available number of replicates was used for each dataset and clearly detailed in the text for each experimental dataset. The number of replicates is in line with literature in the filed, for example two biological replicates were used in the sex sorted Drosophila embryos Hi-C datasets
Data exclusions	No data were excluded from the analysis.
Replication	The manuscript is using previously released genomics datasets. Nevertheless we verified the reproducibility of data between replicates using state of the art methods for literature in this field. Namely, the biological replicates for Hi-C data in male or female sex sorted Drosophila embryos we verified their reproducibility by computing the stratum-adjusted correlation coefficient (SCC) with the hicrep tool. The sex sorted embryos replicates show an SCC value of 0.97 for the male and 0.98 for the female samples.
Randomization	Not applicable
Blinding	Not applicable

Reporting for specific materials, systems and methods

We require information from authors about some types of materials, experimental systems and methods used in many studies. Here, indicate whether each material, system or method listed is relevant to your study. If you are not sure if a list item applies to your research, read the appropriate section before selecting a response.

Materials & experimental systems

n/a	Involved in the study
☒	☐ Antibodies
☒	☐ Eukaryotic cell lines
☒	☐ Palaeontology
☒	☐ Animals and other organisms
☒	☐ Human research participants
☒	☐ Clinical data

Methods

n/a	Involved in the study
☒	☐ ChIP-seq
☒	☐ Flow cytometry
☒	☐ MRI-based neuroimaging
